# Emergence of porcine circovirus‐like viruses associated with porcine diarrheal disease in China

**DOI:** 10.1111/tbed.14223

**Published:** 2021-07-17

**Authors:** Xianhui Liu, Xinming Zhang, Ge Xu, Zhe Wang, Hanqin Shen, Kaiqi Lian, Yihan Lin, Jihao Zheng, Pengshuai Liang, Leyi Zhang, Yanling Liu, Changxu Song

**Affiliations:** ^1^ College of Animal Science & National Engineering Center for Swine Breeding Industry South China Agriculture University Guangzhou P. R. China; ^2^ Wen's Foodstuff Group Co. Ltd Guangdong Enterprise Key Laboratory for Animal Health and Environmental Control Yunfu P. R. China; ^3^ School of Biotechnology and Food Science Anyang Institute of Technology Anyang P. R. China

## Abstract

**Background:**

The circular replication‐associated protein (Rep)‐encoding single‐stranded (CRESS) DNA virus emergence in diverse host has been associated with severe disease. Porcine circovirus‐like virus (Po‐Circo‐like [PCL] virus) is a CRESS DNA virus, the prevalence and pathogenicity of which are rarely studied.

**Methods:**

We obtained two blood samples, four faecal samples, and two intestinal samples from a pig farm suffered from diarrheal disease in the delivery room in September 2020 and attempted to isolate and identify a causative pathogen. Subsequently, only PCL virus was positive, and qRT‐PCR was designed to detect the loading titre of PCL virus. We then initiated a heightened surveillance program on the pathogenicity and epidemiology of PCL virus.

**Results:**

Six PCL virus strains, with severe diarrhoea and haemorrhagic enteritis, have been found in six different pig farms in Guangdong province, China. A multiple sequence alignment of these PCL viruses and bovine circovirus‐like virus/CH showed a similarity of 92.5‐94.8% for the Rep protein, indicating these PCL viruses are highly homologous to Bo‐Circo‐like virus associated with calf diarrhoea. There were striking similarities between the PCL virus and bovine circovirus‐like virus outbreaks in aetiological settings and Genomic sequence. We found that 11.2% (20/178) of diarrhoea samples and 13.3% (6/45) of pig farms were positive for PCL virus, suggesting that PCL virus may have spread widely in Pig farms. Moreover, this article underscores the risk of PCL virus spilling over and adapting to new species.

**Conclusions:**

Porcine circovirus‐like virus was found to be associated with porcine diarrheal disease in China.

## 1 INTRODUCTION

The circular replication‐associated protein (Rep)‐encoding single‐stranded (CRESS) DNA virus possess small genomes, prevalence, and affinity for rolling‐circle replication (Zhao et al., 2019). These are widely found in diverse environments, plant samples, dragonflies and damselflies, mosquitoes, rats, bats, duck, cattle, pigs, dogs, human, turkey, and forest musk deer (Liu et al., 2020). The CRESS DNA viruses has six family members: *Circovidae, Nanoviridae, Smacoviridae, Genomoviridae, Bacilladnaviridae*, *and Geminiviridae* (Zhao et al., 2019), established by the International Committee on the Taxonomy of Viruses (ICTV). A seventh family belonging to CRESS‐DNA viruses was recently recognized by the ICTV with the name of *Redondoviridae* (Abbas et al., 2021).

In addition to seven recognized CRESS DNA viral families, a great deal of novel CRESS DNA viruses needing to be classified by ICTV, and a group of viruses with large genomes (2833‐3923 bp), according to genomic and Rep‐phylogenetic characteristics, have been proposed family *Kirkoviridae* (Guo et al., 2018; Li et al., 2015; Shan et al., 2011). Moreover, all the Rep proteins of the viruses belonging to proposed family *Kirkoviridae* indicate significant genetic distance with those of the viruses within family *Circoviridae* (Guo et al., 2018; Shan et al., 2011; Sun et al., 2020).The family *Circovidae* includes two genera: Cyclovirus and Circovirus. PCV‐associated disease (PCVAD) is associated to PCV2 (porcine circovirus 2) infection (Meng, 2013), causing great harm to the pig industry. In recent years, Porcine‐like virus P1, PCV3, and PCV4 have been reported to be found in pigs (Opriessnig et al., 2020; Sun et al., 2020; Wen et al., 2018).

In 2011, a novel pig‐derived CRESS DNA virus was discovered in pig faces and named PCL virus (porcine circovirus‐like virus) (Shan et al., 2011). A novel CRESS DNA virus named Bo‐Circo‐like virus from a calf with severe haemorrhagic enteritis has recently been detected in China (Guo et al., 2018), and three PCL viruses in pigs with diarrhoea have been found in Guangxi, China (Sun et al., 2020). The PCL virus belongs to proposed family *Kirkoviridae*, the prevalence and pathogenicity of which are rarely studied.

Porcine diarrhoea is one of the primary causes of piglet death, which results in severe nutrition absorption and slow growth in pigs, leading to substantial economic losses. Newborn piglets are highly vulnerable to some enterovirus infections, causing severe diarrhoea, enteritis, and vomiting. We obtained two blood samples, four faecal samples, and two intestinal samples from a pig farm suffered from diarrheal disease in the delivery room in September 2020 and attempted to isolate and identify a causative pathogen. Then, we identified microbial pathogens associated with diarrhoea, and only PCL virus was detected in the diarrhoea samples. Subsequently, we initiated a heightened surveillance program on the pathogenicity and epidemiology of PCL virus.

## 2 MATERIALS AND METHODS

### 2.1 Clinical samples collection

In this study, 178 clinical samples with severe haemorrhagic enteritis, diarrhoea, lymphadenopathy, loss of appetite, and vomiting of piglets, including intestinal tissue or faces, were obtained from 45 swine farms in different regions of Guangdong province, China from April to December 2020 and stored at −80°C.

### 2.2 DNA/RNA extraction of viruses

Clinical samples obtained were grounded with double resistance PBS and repeatedly freeze‐thawed three times. The viral DNA/RNA is then extracted using RaPure Viral RNA/DNA Kit (Magen, R4410‐3, China).

### 2.3 PCR array

The primers of porcine enteroviruses, including PEDV, PDCoV, TGEV, RV, SADS‐CoV, PTV, PKV, PBV, PBoV, SaV, PSV, NOV, and PCV4, have been found from the literature (Li et al., 2012; Li et al., 2019; Meng et al., 2018; Sun et al., 2020; Shen et al., 2012; Sun et al., 2020; Xue et al., 2018; Wang et al., 2017; Zhou et al., 2019; Zhang et al., 2020). Five prime pairs were designed based on the reference sequence of the PCL virus 21 and 22 strain determined in the United States and the PCL virus GX14, GX15, and GX19 detected in China (Supporting information Table S1). PCR products were first isolated and identified by agarose gel electrophoresis, and then obtained using a gel recovery kit and cloned into a blunt‐T plasmid (Takara). Whereas the ligands were transformed into DH‐5α cells for gene cloning. The positive clones screened by PCR were sent to a commercial facility (Sangon Bioengineering Co., Ltd, China) for sequencing.

### 2.4 Real‐time PCR array

To investigate tissue tropism of PCL virus in diarrheal piglets, a SYBR green quantitative real‐time PCR (qRT‐PCR) targeting the conservative regions of PCL virus was developed based on PCL virus CQY09. PCR arrays for PCL virus in different tissues of two 7‐day‐old piglets with diarrhoea were performed with Eastep qPCR Master Mix (Promega, China) on an ABI 7500 Real‐Time PCR System. Detailed information on gene primers used in qRT‐PCR is listed (Supporting information Table S1). The results from three independent tests were analysed using GraphPad Prism 5.0 software.

### 2.5 Phylogenetic analysis

The complete gene sequences of PCL viruses obtained in this study have been uploaded to Genbank with the accession numbers (Supporting information Table S2). The genome lines were assembled using Lasergene. Subsequently, all arrangements were further aligned with MegAlign (Lasergene) using the ClustalW alignment method. A phylogenetic tree was built using the maximum likelihood method with 1000 bootstrap replicates in MEGA7 software.

## 3 RESULTS AND DISCUSSION

CRESS viruses can infect a wide range of animals, even plants and mosquitoes. These viruses are found in pigs include PCV1, PCV2, PCV3, PCV4, porcine circovirus‐like virus P1 and PCL virus (Meng, 2013; Ouyang et al., 2019; Wen et al., 2018; Zhang et al., 2020). PCV2 have been associated with clinical diseases in pig farms known as PCV‐associated disease (PCVAD), causing substantial economic losses (Meng, 2013). At present, PCV2 and PCV3 are widely popular in the global pig industry (Meng, 2013; Opriessnig et al., 2020). PCL virus is very similar to PCV, and both have a circular genome, however, PCL virus does not have a typical capsid protein (Cap). The epidemiology of PCL virus has rarely been reported in China.

**FIGURE 1:**
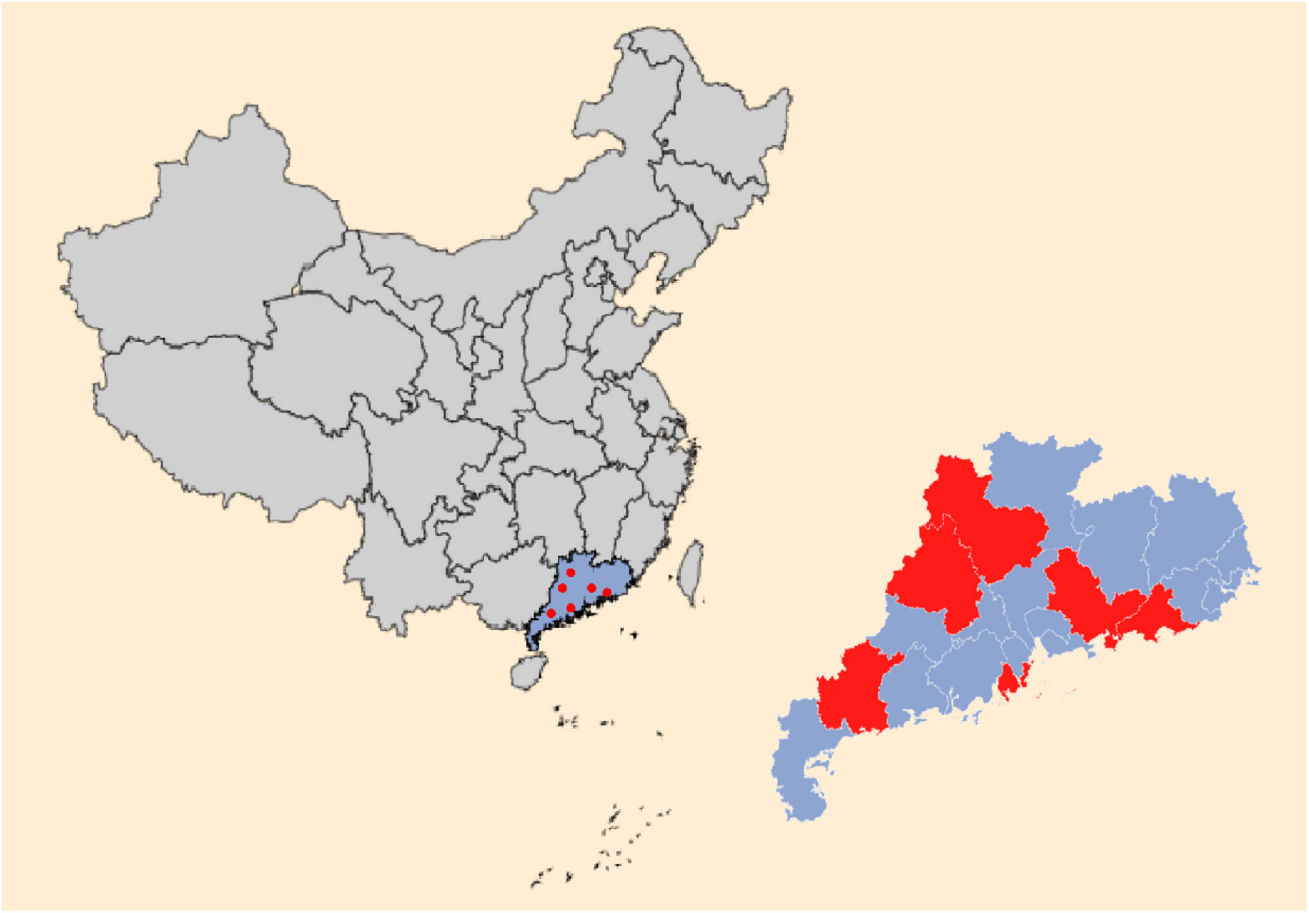
Geographic distribution of pigs with Porcine circovirus‐like virus in China. Areas where surveillance of Porcine circovirus‐like virus was carried out are shown in blue. Red circles indicate the locations of farms with confirmed Porcine circovirus‐like virus infection.

In this study, PCL virus was detected from six pig farms in different areas (Figure 1). This study found that 11.2% (20/178) of diarrhoea samples and 13.3% (6/45) of pig farms were positive for PCL virus, only indicating that PCL virus has been evenly and widely prevalent in Guangdong Province (Figure 1), not indicating the transmission ability and epidemiology of PCL virus. More research on the prevalence of PCL virus in different areas and seasons are needed.

In the six farms, intestinal contents did not identify any pathogenic bacteria causing diarrhoea in pigs. In farm B, the PCL virus isolate CQY09 were detected from newborn piglets (1 week to 4 weeks) associated with severe diarrhoea, haemorrhagic enteritis, and vomiting in Qingyuan, China. The swine enterovirus (PEDV, PDCoV, TGEV, RV, SADS‐CoV, PTV, PKV, PBV, PBoV, SaV, PSV, NOV and PCV4) have been retested on PCL positive samples as shown in Supporting information Table S3, indicating only PCL virus was positive, and the highest copy number of PCL virus in all detected tissues of two 7‐day‐old piglets infected with PCL virus was found in the faces (Supporting information Figure S1). Anatomy of diseased piglets found small intestine mucosa abscission, and intestinal mucosal lymph node enlargement (Supporting information Figure S1). There was a higher incidence of morbidity, while lower mortality among piglets as a result of relief of symptoms by breeder's saline rehydration used in piglets with diarrhoea. Moreover, the PCL virus isolate CSW10 were detected in piglets aged 2 weeks at a farm in Shanwei (farm D), Guangdong province, and no other enteroviruses were detected in the samples (Supporting information Table S3). Although there was a serious outbreak of diarrheal disease on the farm, further investigation was not conducted due to the strict control of the farm.

Further studies shown that PCL positive sample CMM06 in farm A was coinfected with PBOV. Enteritis and diarrhoea primarily occurred in piglets, but severe diarrhoea, loss of appetite, and significant reduction in average daily weight gain occurred in fat pigs, speculating the cause of coinfection of these viruses. Besides, three farms of the six PCL virus‐positive farms were coinfected with porcine epidemic diarrhoea virus. Although piglets with diarrheal diseases in the six farms have higher morbidity (80‐100%) and mortality (20‐70%), this may only be caused by PEDV, PBoV or PCL virus, or PCL virus may aggravate PEDV or PBoV infection. The detection of PCL virus was closely associated with diarrhoeal disease in pigs in this study, the presence of the virus in healthy pigs was not assessed. The PCL virus has not been successfully isolated from cells, so animal model tests on the pathogenicity of PCL virus cannot be carried out. In conclusion, more studies on the pathogenicity of PCL virus are needed.

**FIGURE 2:**
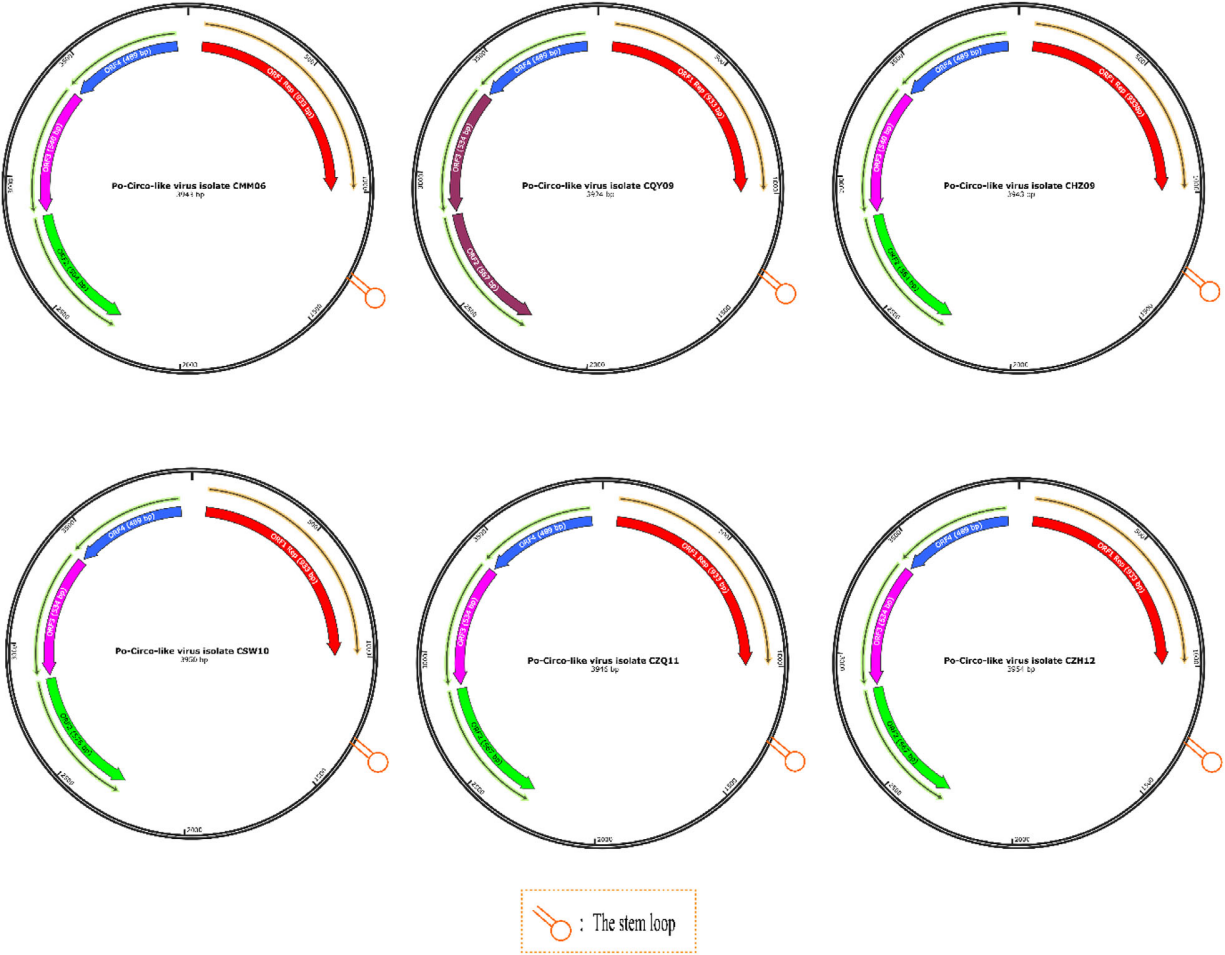
Predicted genome organization of six Po‐Circo‐like virus strains.

Subsequently, the full genomes of six strains were sequenced, and their gene characteristics were further analysed (Figure 2). The genomes of these strains were all circular, with a Rep protein and a stem loop of the same length (Figure 2). The six PCL virus strains contains 3924, 3943, 3946, 3950, and 3954 nucleotides in length (Supporting information Table S2), which differ with the PCL virus GX14 (3944 bp), GX15 (3944 bp), and GX19 (3944bp) detected in China, the PCL virus 21 (3921bp) and 22 (3922 bp) detected in the United States, and Bo‐Circo‐like virus/CH (3902 bp) discovered in China. PCVs have a stem‐loop structure (Cheung, 2007), whereas PCL virus contains a 14‐nucleotide stem loop (Sun et al., 2020), which is essential for replicating viruses. Stem loop of six PCL virus includes GGGCAA **T** TCTGCCC, GGGCAA **G** TCTGCCC, and GGGCAA **A** TCTGCCC (Supporting information Table S2). Moreover, the substitution effects in the loop of the PCL virus (T, G, and A) on pathogenicity, replication, and infectivity require further study.

**FIGURE 3:**
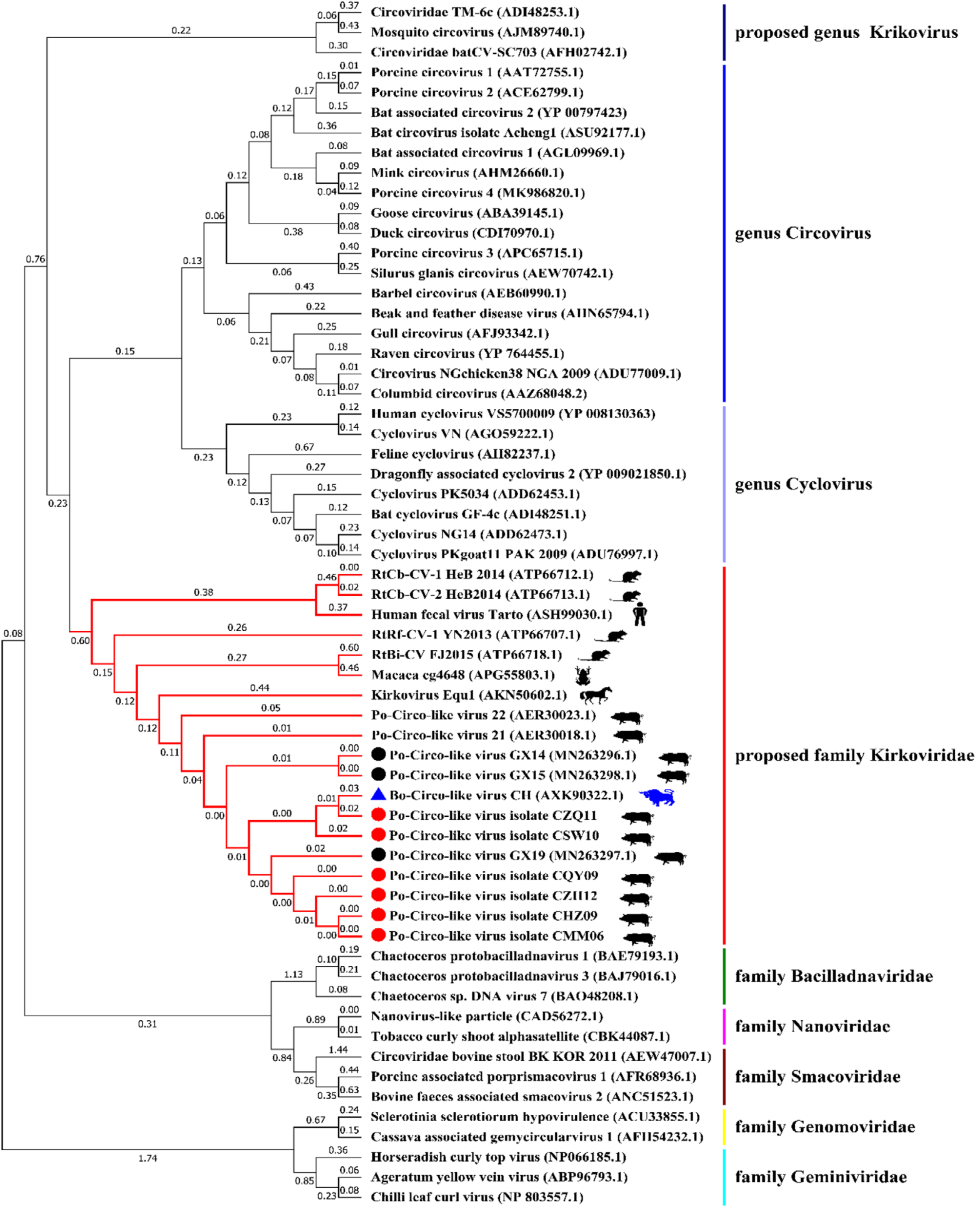
Phylogenetic tree was constructed based on amino acid sequence of the Rep protein. The coding regions for the Rep protein were analysed by means of the neighbour‐joining method using Poisson correction and complete deletion of gaps. Bootstrap testing (1000 replicates) was performed, and the bootstrap values were indicated. The strains in the present study are marked with red circles. Black circles indicate three reported strains of the Po‐Circo‐like virus in Guangxi, China. The Bo‐Circo‐like virus strain is marked with blue triangle.

The genome‐wide pairwise identities between the six PCL virus strains and the Bo‐Cir‐like virus were about 89%. Besides, a multiple sequence alignment of these strains and Bo‐Circo‐like virus CH, showed a sequence similarity of 86.2‐94.4% for the rep gene lines and sequence similarity of 89.4‐97.7% for the Rep protein arrangements. Phylogenetic trees based on amino acid sequences of the Rep protein have been constructed (Figure 3), indicating that the six PCL virus strains were highly homologous to bovine circovirus‐like virus associated with calf diarrhoea in China.

**FIGURE 4:**
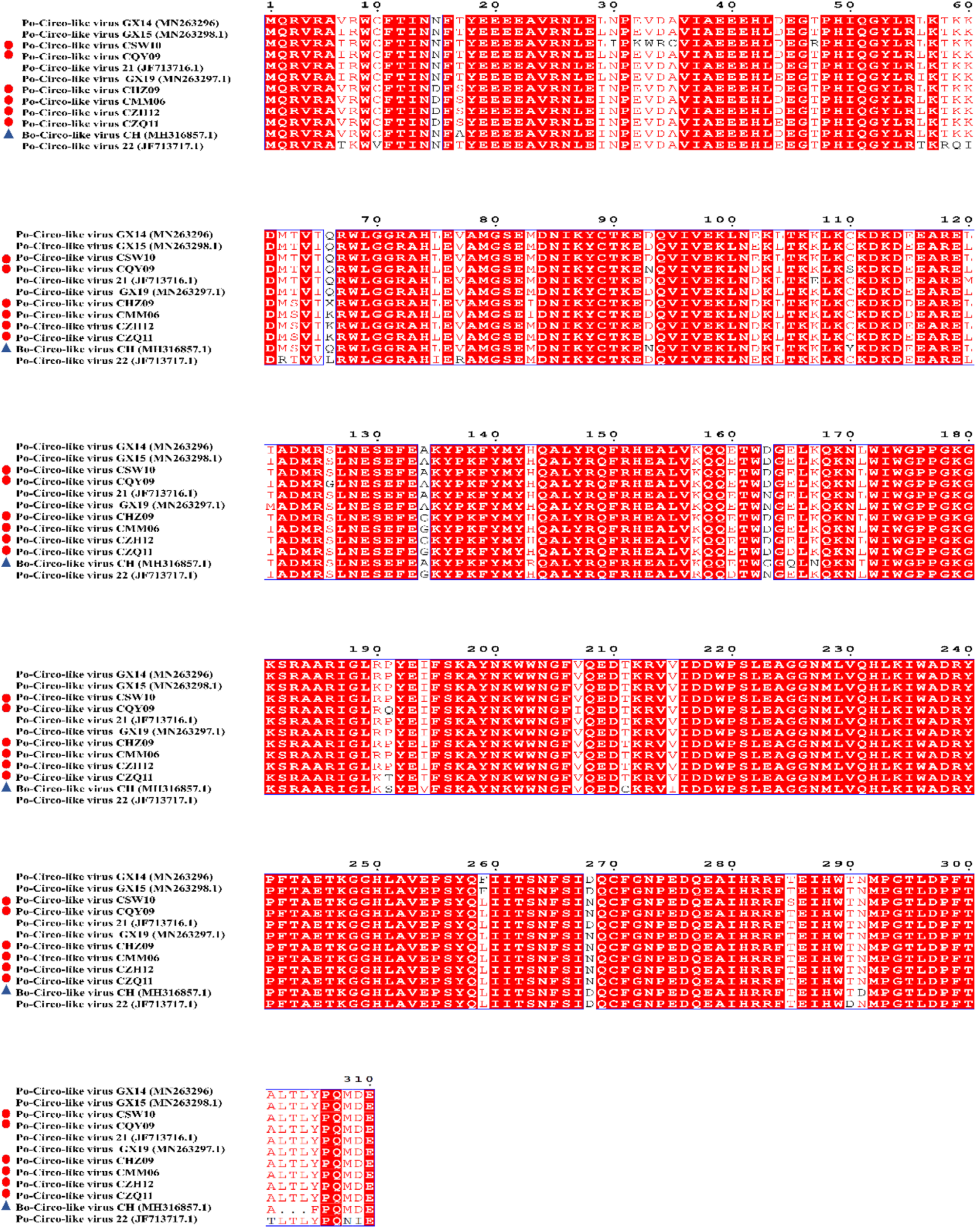
Amino acid comparison and analysis on Rep protein. The strains in the present study are marked with red circles. Blue triangle indicates the Bo‐Circo‐like virus, and Unlabelled strains indicate three strains of the Po‐Circo‐like virus detected in China and two strains of the Po‐Circo‐like virus found in the United States.

The strains of PCL virus had a close relationship to strains of Bo‐Circo‐like virus CH and the strains of GX14, GX15, GX19, 21 and 22 of the PCL viruses on amino acid sequences of the Rep protein (Figures 3 and 4), indicating that all these viruses were most likely to belong to the same species. Fewer amino acid mutations on the Rep protein of these viruses were observed (Figure 4), the substitution effects on pathogenicity, replication, and infectivity require further investigation.

The striking similarities between the PCL virus and bovine circovirus‐like virus outbreaks in aetiological settings and Genomic sequence cannot be ignored (Guo et al., 2018), suggesting that these strains may be the same virus and infect different hosts, also underscoring the risk of PCL virus spilling over and adapting to new species.

These findings have helped us to understand the status of intestinal infection in the Chinese pig population and also prompted us to accelerate research into pathogenesis and epidemiology of the PCL virus.

## CONFLICT OF INTEREST

The authors declare no conflict of interest.

## DATA AVAILABILITY STATEMENT

The data that support the findings of this study are available from the corresponding author upon reasonable request.

## CODE AVAILABILITY

The authors show that it is available.

## ETHICS APPROVAL

This work is approved.

## CONSENT TO PARTICIPATE

The authors approve that the study is suitable for participation.

## CONSENT FOR PUBLICATION

The authors agree that the study is publishable.

## AUTHORS' CONTRIBUTIONS

Conceived and designed the experiments: CX S. Performed the experiments:XH L, XM Z, G X, Z W, HQ S, and KQ L. Analysed the data: XH L, JH Z, YH L, PY L, LY Z and YL L. Contributed reagents/materials/analysis tools: CX S. Worte the paper: XH L and CX S.

## Supporting information

SUPPORTING INFORMATION
