# Supplementary material for: Emergence of porcine circovirus‐like viruses associated with porcine diarrheal disease in China
Source: Transbound Emerg Dis. 2021 Jul 17;68(6):3167–73. doi: 10.1111/tbed.14223 (PMC9290044; doi:10.1111/tbed.14223)
Supplement: Supplementary file 1 — SUPPORTING INFORMATION [file TBED-68-3167-s001.docx]

**Supplementary file Table 1** Primers used for PCR amplification in the present study.

| **Primer name** | **Primer sequence (5^,^ -3^,^)** | | **Size (bp)** |
| --- | --- | --- | --- |
| **PCL-3754F** | **TAGGGATTTCCGCTTGGATCAAGTACT** | **1370** | |
| **PCL-1182R** | **TATTACCTTTAGCGGAATCAAAATCGGAC** |  | |
| **PCL-963F** | **ATGCCTGGCACCTTAGACCCCTTTA** | **916** | |
| **PCL-1879R** | **AATGAACTGACCACTCATGAA** |  | |
| **PCL-1833F** | **GTTAACCGCAATTGAATTTTGA** | **1179** | |
| **PCL-3012R** | **TGGCAGGCGTATGGCCAGGTGATCT** |  | |
| **PCL-2830F** | **GTACGATGAGGATCAAACATTAA** | **1160** | |
| **PCL-46R** | **CGCACTCTTTGCATTTTGATTAATGAATT** |  | |
| **PCL-D-F (3331)** | **TTAACACGGCGAGTATATCTACCAGA** | **548** | |
| **PCL-D-R (3879)** | **GTCTCAGGACTTCGATGTATTCGACCCTT** |  | |
| **PCL-RT-F (3582)** | **CTGCAAAGGAGACGTCATGG** | **204** | |
| **PCL-RT-R (3785)** | **ACGAGACCCAAATCACCCAA** |  | |

**Supplementary file Table 2** List of the Po-Circo-like virus detected in farms in Guangdong, China

| **Farm** | **The name of Po-Circo-like virus strain** | **the full genomes of virus (bp)** | **Stem loop** | **Genbank number** | **Time**  **(Month-Year)** | **Location** | **Sample types** | **Number of positive samples** |
| --- | --- | --- | --- | --- | --- | --- | --- | --- |
| **A** | **CMM06** | 3943 | GGGCAATTCTGCCC  1330-1343 | MW881205 | **Jun. 2020** | **Maoming** | **3 fecal sample** | **2/3** |
| **B** | **CQY09** | 3924 | GGGCAAGTCTGCCC  1322-1335 | MW881206 | **Sep. 2020** | **Qingyuan** | **4 fecal sample, 2 blood sample and 2** **intestinal sample** | **7/8** |
| **C** | **CHZ09** | 3943 | GGGCAATTCTGCCC  1330-1343 | MW881207 | **Sep. 2020** | **Huizhou** | **2 intestinal sample and 2 fecal sample** | **3/4** |
| **D** | **CSW10** | 3950 | GGGCAAGTCTGCCC  1334-1347 | MW881208 | **Oct. 2020** | **Shanwei** | **2 fecal sample and 2 fecal swabs** | **3/4** |
| **E** | **CZQ11** | 3946 | GGGCAAATCTGCCC  1322-1335 | MW881209 | **Nov. 2020** | **Zhaoqing** | **3 fecal sample and 1 intestinal sample** | **3/4** |
| **F** | **CZH12** | 3954 | GGGCAAGTCTGCCC  1330-1343 | MW881210 | **Dec. 2020** | **Zhuhai** | **3 fecal sample and 1 intestinal sample** | **2/4** |

Faeces, intestine or faecal swabs collected from April to December 2020 were tested. Sampling type and number, time and location of samples per farm were collated.

**Supplementary file Table 3** List of all known swine viruses tested by PCR on the six Po-Circo-like virus positive farms with outbreaks of diarrheal disease.

| **Farm** | **PEDV** | **TGEV** | **SADS-CoV** | **PDCoV** | **RV** | **PBoV** | **PBV** | **PTV** | **PKV** | **PSV** | **PCV4** | **Norvorirus** | **CSFV** | **FMDV** | **ASFV** | **PRV** | **PRRSV** | **PCV2** | **PPV** | **PCV3** |
| --- | --- | --- | --- | --- | --- | --- | --- | --- | --- | --- | --- | --- | --- | --- | --- | --- | --- | --- | --- | --- |
| **A** | **-** | **-** | **-** | **-** | **-** | **√** | **-** | **-** | **-** | **-** | **-** | **-** | **-** | **-** | **-** | **-** | **-** | **-** | **-** | **-** |
| **B** | **-** | **-** | **-** | **-** | **-** | **-** | **-** | **-** | **-** | **-** | **-** | **-** | **-** | **-** | **-** | **-** | **-** | **-** | **-** | **-** |
| **C** | **√** | **-** | **-** | **-** | **-** | **-** | **-** | **-** | **-** | **-** | **-** | **-** | **-** | **-** | **-** | **-** | **-** | **-** | **-** | **-** |
| **D** | **-** | **-** | **-** | **-** | **-** | **-** | **-** | **-** | **-** | **-** | **-** | **-** | **-** | **-** | **-** | **-** | **-** | **-** | **-** | **-** |
| **E** | **√** | **-** | **-** | **-** | **-** | **-** | **-** | **-** | **-** | **-** | **-** | **-** | **-** | **-** | **-** | **-** | **-** | **-** | **-** | **-** |
| **F** | **√** | **-** | **-** | **-** | **-** | **-** | **-** | **-** | **-** | **-** | **-** | **-** | **-** | **-** | **-** | **-** | **-** | **-** | **-** | **-** |

The dash shows a negative PCR result. The red tick indicates a positive test. PEDV, porcine epidemic diarrhoea virus; TGEV, porcine transmissible gastroenteritis virus; SADS-CoV, swine acute diarrhoea syndrome coronavirus; PDCoV, porcine deltacoronavirus; RV, porcine rotavirus; PBoV, porcine bocavirus; PBV, porcine picobirnavirus; PTV, porcine teschovirus; PKV, porcine kobuvirus; PSV, porcine sapelovirus; PCV4, porcine circovirus type 4; CSFV, classical swine fever virus; FMDV, foot and mouth disease virus; ASFV, African swine fever virus; PRV, Pseudorabies virus; PRRSV, porcine reproductive and respiratory syndrome virus; PCV2, porcine circovirus type 2; PPV, porcine parvovirus; PCV3, porcine circovirus type 3.


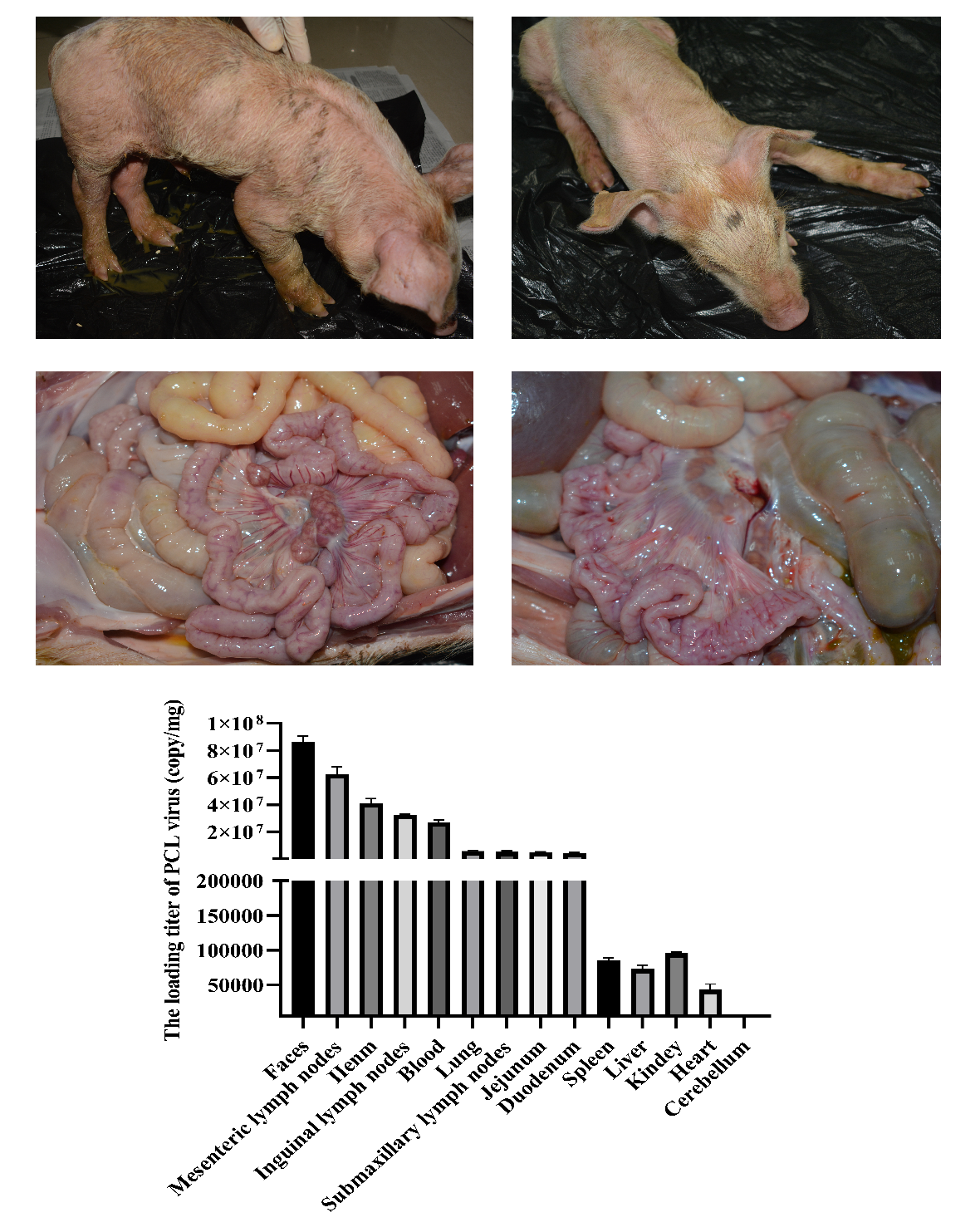


**Supplementary file Fig 1.** Tissue distribution of the Porcine circovirus-like virus strains CQY09 in different tissues of two seven-day-old piglets with diarrhea on farm B.
